# Pre-Antibiotic Treatment Followed by Prolonged Repeated Faecal Microbiota Transplantation Improves Symptoms and Quality of Life in Patients with Irritable Bowel Syndrome: An Observational Australian Clinical Experience

**DOI:** 10.1155/2022/6083896

**Published:** 2022-10-13

**Authors:** Harrison Hamblin, Anoja W. Gunaratne, Annabel Clancy, Denise Pilarinos, Antoinette LeBusque, Marie Vic M. Dawson, Thomas J. Borody

**Affiliations:** Centre for Digestive Diseases, Five Dock, NSW, Australia

## Abstract

**Background:**

The use of faecal microbiota transplantation (FMT) in irritable bowel syndrome (IBS) has frequently failed to induce long-term symptomatic improvement. The use of multiple FMT infusions is one proposed mechanism through which the efficacy of FMT can be amplified.

**Aims:**

To evaluate the safety and efficacy of a novel six-month FMT treatment protocol in IBS.

**Methods:**

Patients diagnosed with IBS confirmed by Rome IV Criteria were recruited for single-centre, single-arm, prospective clinical observational study. Participants received one colonoscopically delivered FMT followed by 36 rectal enemas across a six-month period. Validated abdominal symptoms and Short-Form (SF-36) Quality of Life (QOL) questionnaires were collected at baseline, week-12, week-24, and week-56, respectively. Wilcoxon matched-pairs signed-rank tests were conducted to compare differences in abdominal symptom and SF-36 QOL scores over the follow-up timepoints. Statistical significance was set at 5%.

**Results:**

Sixty participants diagnosed with IBS [IBS-constipation (*n* = 27), IBS-diarrhoea (*n* = 18), and IBS-mixed (*n* = 15)] received the six-month FMT treatment. IBS symptom severity reduction was achieved in up to 61% of respondents at week-12, 64% of respondents at week-24, and maintained in up to 75% of respondents at week-52. Long-term reduction in symptom severity was associated with an increase in QOL, achieved in up to 64% of respondents at week-52 when compared to baseline. Adverse events were experienced in 28% of participants, though they were both transient and mild in nature.

**Conclusions:**

Six-month sustained FMT appears to be both safe and effective in the short- and long-term alleviation of IBS associated symptoms as well as improving participant QOL.

## 1. Introduction

Irritable bowel syndrome (IBS) is a chronic disease that affects more than 10% of the global population, making it the most common functional gastrointestinal disorder worldwide [1]. It is more commonly experienced by females and is typically associated with recurrent abdominal pain related to defecation and alterations in either stool form or frequency [2, 3]. Current diagnostic approaches include an assessment using the Rome IV Criteria, which under recent versions has introduced IBS subtyping based on the stool consistency predominantly experienced by the patient; constipation (IBS-C), diarrhoea (IBS-D), or mixed type (IBS-M) [4]. Despite these diagnostic advancements, understanding of the pathophysiology of IBS and its various subtypes remains elusive [5]. Consequently, current pharmacologic and dietary approaches, such as restricting foods with highly fermentable oligosaccharides, disaccharides, monosaccharides, and polyols (FODMAPs), are aimed at providing symptomatic relief rather than treating an underlying cause [6]. As a result, such approaches provide inadequate symptom reprieve in the long term, which when combined with the increasing prevalence of IBS in recent years has resulted in a significant economic impact and burden of disease [7–10].

The gut microbiome, a combination of the resident microflora and their metabolic products of digestion, has recently been implicated in the pathogenesis of IBS due to an abundance of research reporting reduced diversity in the intestinal microflora of individuals with IBS when compared to healthy controls [11–13]. In response, several therapies aimed at restoring this dysbiosis have been trialled with varying success. Probiotic supplementation has been suggested to promote gut flora health, although randomised controlled trials (RCTs) aimed at investigating their role in the treatment of IBS have produced inconsistent outcomes due to a lack of knowledge surrounding the optimal type and dosage [14, 15]. Dietary supplements and modifications are yet another mechanism through which the microbial composition of the gut can be altered. Indeed, due to their reported bifidogenic effect, it was posited that dietary fibre in the form of prebiotics could provide benefit to patients with IBS, where reduced luminal and mucosal *Bifidobacterium* is commonly observed [12, 16]. Studies assessing the role of prebiotics in IBS however, typically report no significant difference in improvement when compared with placebo, and exacerbation of existing symptoms, such as bloating are frequently reported by patients [12, 17, 18].

In recent years, interest in the therapeutic role of faecal microbiota transplantation (FMT) in chronic gastrointestinal conditions, such as Inflammatory Bowel Disease (IBD) and IBS has increased due to its reported success in the treatment of recurrent *Clostridioides difficile* infection (rCDI) [19]. FMT involves the transfer of healthy donor stool and its constituents into the colon of unwell patients, such as those with IBS. It is hypothesised that the intestinal dysbiosis observed in such patients could be reversed by FMT, thus resulting in improvements in disease. Very positive initial results for FMT were reported in a number of small single-arm trials (SATs) in IBS [20–23]. Larger RCTs which have examined the role of FMT delivered via single colonoscopic/gastroscopic infusions have demonstrated in IBS both symptomatic improvement and gut microbial compositional changes when compared with placebo [24–27]. However, oral encapsulated forms have frequently displayed no significant differences [28–30], demonstrated in a recent meta-analysis [31]. Consequently, the burden of disease remains high in patients with IBS, highlighting the need for improvements in available therapeutic options.

One proposed mechanism through which the efficacy of FMT can be improved is by increasing the dosage or the number of infusions a patient receives [32–34]. Indeed, recent research into the treatment of acute infections, such as rCDI and IBDs namely ulcerative colitis and Crohn's disease have shown an increased efficacy following multiple FMT infusions [35–38]. However, such research is currently lacking in IBS, with only one small study examining the effects of multiple infusions on patients. Importantly, the study in question reported that 70% of those who failed to respond to an initial 30 g FMT infusion displayed significant improvement following a subsequent 60 g infusion into the duodenum via gastroscopy, suggesting that dosing and repeated infusions may have an effect on the efficacy of FMT treatment in IBS [27]. In addition to the paucity of data on the role of repeated FMT in IBS, long-term information on the sustainability of the improvements in symptoms it may provide are also lacking. Therefore, this study aimed to examine the safety and efficacy of a novel six-month sustained FMT infusion protocol used at our centre in patients with IBS over a period of 12 months.

## 2. Materials and Methods

### 2.1. Study Design

This was a single-arm, prospective, single-centre clinical observational study conducted at The Centre for Digestive Diseases, Five Dock, NSW, Australia, between May 2018 and August 2020. This study was approved by the Centre's independent Human Research Ethics Committee (HREC), study number: CDD19/C02.

### 2.2. Clinical Procedures

Antibiotics followed by FMT has been a standard treatment for IBS at our centre for over 30 years. In 2018 a novel six-month home FMT program was implemented at our centre. The standard clinical procedures are outlined below.

### 2.3. Participant and Home FMT Donor Pre-Treatment Screening

Prior to the commencement of the extended home FMT program, participants were examined and screened by a clinical nurse and their attending gastroenterologist to rule out any other acute infections or chronic diseases. All included participants were given the opportunity to discuss alternative treatment options with their physicians. In addition, due to the prolonged nature of the treatment, participants were instructed to recruit a home FMT donor. This person was required to provide regular donations to the participant's residence during the six-month treatment period (Table 1). Potential home donors were screened according to both national and international standards [39, 40].

### 2.4. Pre-Treatment Antibiotics

Once a suitable home donor had passed standard donor screening protocols [39, 40], eligible participants began pre-treatment antibiotics consisting of a combination of vancomycin and/or rifaximin and/or tinidazole and/or metronidazole for a minimum of two weeks and up to a maximum of three months prior to commencement of FMT, individualised to the patient by the treating physician. The pre-treatment antibiotic combinations were personalised in cases of allergic reactions, drug sensitivities, or a lack of response to the first-line combinations. All participants were instructed to cease pre-treatment antibiotics two days prior to the commencement of FMT.

### 2.5. Six-Month Extended FMT Protocol

Participants commenced the six-month FMT protocol at our centre using FMT derived from local registered donors. During the study period, our centre had 13 active stool donors who were regularly screened in accordance with Australian and international guidelines [39, 40]. Within four hours of defecation, donor stool was weighed, pooled together, and then blended using a 2000 w blender with isotonic saline (0.9% sodium chloride). For every 1 g of stool, 4 ml of 0.9% sodium chloride was added to produce a homogenate solution. The homogenate was then passed through a 330 μm filter to remove undigested food material. On day 1 of the initial ten-day FMT treatment performed at our centre, six, 50 ml syringes were filled with the homogenate solution and infused directly into the caecum of each participant during a sedated colonoscopy (Table 1). On days 2–10, 300 ml of FMT was delivered once per day via rectal retention enemas at our centre by trained clinical nurses (Table 1). Participants were instructed to retain these infusions for a minimum of four hours and were offered loperamide to assist with this. All infusions at our centre were stored at room temperature until used, which occurred within four hours of stool processing.

On the final day of the ten-day initial treatment at our centre, participants attended a home FMT training session where they were provided with a home FMT kit consisting of a 300 w blender, 32 enema bags, 32 1L bottles of 0.9% isotonic sodium chloride, and a re-usable 330 μm filter, to enable the continuation of infusions using their home donor for the remaining 22 weeks of treatment whilst at home (Table 1). Consistent with the fresh FMT protocols used at our centre, participants were instructed to process their home donors stool donations within four hours of defecation and to perform the 300 ml rectal enema infusion no longer than four hours after stool processing. Participants were also provided with hand hygiene training and both visual and written demonstrations on how to perform FMT infusions at home safely. Nurse practitioners were available if participants required additional support whilst completing FMT at home.

### 2.6. Dietary Considerations and Mid-Treatment Screening

During the six-month FMT treatment period, both participants and their home donors were instructed to commence a high fibre diet along with three different prebiotic fibre supplements, such as apple pectin, inulin, and *N*-acetyl-glucosamine, known to promote short-chain fatty acid (SCFA) production in the gut [41, 42], and avoid raw and processed animal products to reduce the risk of food-borne bacterial infection [43]. Whilst continuing treatment at home, participants' home donors were subject to regular stool and blood screening every one and three months, respectively [39, 40]. Participants were physically examined by a clinical nurse at 12 and 24 weeks of treatment and by their physician 52 weeks after the commencement of FMT.

### 2.7. Participants

For evaluation, eligible participants included those with a confirmed diagnosis of IBS; as established by the Rome IV Criteria. Participants with controlled coeliac disease (stable on a gluten-free diet with normal serology) were included. Participants were excluded if they were less than 18 years of age at the time of treatment, had IBD, or failed to provide written informed consent. Participants were consecutively enrolled as part of standard clinical treatment between March 2018 and ceased in August 2019 and one year follow up continued to August 2020. The sample size was calculated based on a required minimum sample of *N* = 31 for pre- and post-statistical testing (*α* (two-tailed) = 0.5, *β* = 0.2, moderate effect size = 0.5, and standard deviation = 1.0) [44] with an assumed minimum follow-up rate of 60% [45].

### 2.8. Data Collection

Participants were instructed to complete validated abdominal symptoms [46] and Short-Form 36-Item Quality of Life (SF-36 QOL) [47] questionnaires at baseline, week-12, week-24, and week-52, respectively, following the commencement of FMT. The abdominal symptoms questionnaire used in this study was selected over other, more widely used measures of symptomatic improvement in IBS, such as the IBS-Symptom Severity Score (IBS-SSS) [48] as it includes a broader range of symptoms such as heartburn, which is becoming increasingly associated with the disease. The SF-36 QOL questionnaire was included as a means of evaluating the emotional and psychological fluctuations of participants in response to both symptomatic changes and the nature of the FMT protocol used. For logistical reasons, the baseline questionnaires were provided to participants during their home FMT education session which occurred two weeks after their first FMT infusion. Participant baseline demographic data was collected which included; age, sex, IBS subtype, relevant comorbidities, and pre-treatment antibiotic combination and duration. Concomitant medications prescribed throughout the six-month treatment period were also recorded along with any adverse events experienced by participants. Adverse events were graded using the Common Terminology Criteria for Adverse Events (CTCAE) version 4.0 [49]. For participants who did not complete the entire six-month treatment, the total duration was recorded along with the cause of cessation. All collected patient data was de-identified and entered into a Microsoft Excel file. Participants were contacted via phone and email with reminders to complete follow-up questionnaires in order to reduce response bias.

### 2.9. Outcomes

Primary outcome measures were both a short-term and long-term reduction in the severity of IBS associated abdominal symptoms and improvement in participant QOL assessed at both 12 and 24 weeks through to week-52 by the abdominal symptoms and SF-36 QOL questionnaires [50, 51]. Secondary outcome measures included; the safety of six months sustained FMT, assessed by the CTCAE, and differences in both short and long-term improvements between IBS subtypes.

### 2.10. Statistical Considerations

Basic descriptive statistics, such as the median and range were performed in Microsoft Excel. All other statistical analysis was performed on GraphPad Prism Eight, version 8.4.3 for Windows, GraphPad Software, San Diego, California, USA, http://www.graphpad.com. Statistical significance was set at 0.05. Kruskal–Wallis and chi-squared analysis was performed to identify differences in baseline demographic variables such as age, sex, comorbidities, and pre-treatment antibiotic combinations between IBS subtypes. Chi-squared tests were also used to detect any differences by IBS subtype in the prescription of additional medications throughout treatment and the rate of adverse events experienced by participants. Differences in abdominal symptom and SF-36 QOL scores between IBS subtypes at each timepoint: baseline, week-12, and week-24 were assessed by Kruskal–Wallis analysis. Wilcoxon matched-pairs signed-rank tests were conducted to compare the differences in abdominal symptom and SF-36 QOL scores over time for both the total cohort and IBS subtypes. Paired *t*-tests were also selected in order to account for missing data points. Independent *t*-tests were used to compare the SF-36 QOL scores of respondents at each timepoint; baseline, week-12, and week-24, against the healthy Australian population norms [49]. Due to the low questionnaire follow-up rate at week-52, only Wilcoxon matched-pairs signed-rank tests were performed at this time point to compare the baseline and week-52 scores for the total cohort in both questionnaires. Normality was determined by the D'Agostino-Pearson test, where all data was found to be non-parametric. As such, all statistical tests used non-parametric approaches except the comparison of SF-36 QOL scores in the total cohort against the healthy Australian population norms. A non-parametric Mann–Whitney *U* test was not used in this instance due to the large sample size of the healthy Australian population data [52].

## 3. Results

### 3.1. Baseline Characteristics

**Figure 1:**
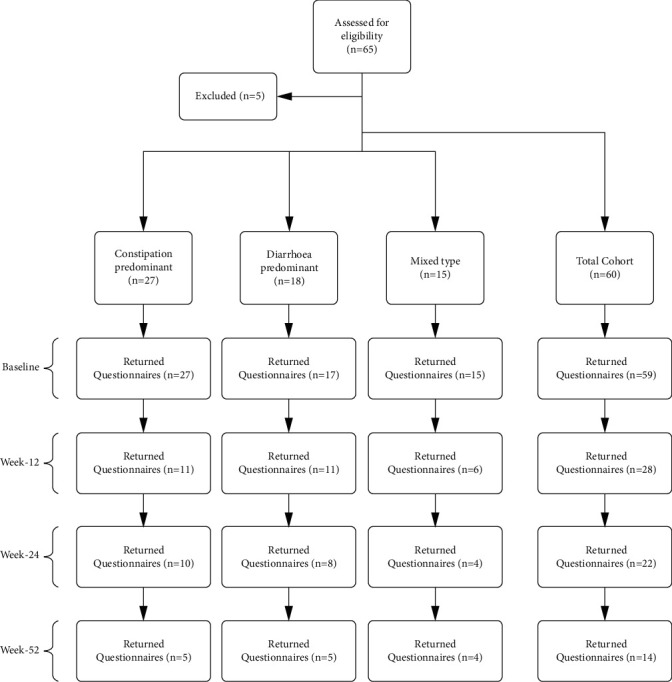
Participant inclusion and questionnaire response rate flowchart for the total cohort and IBS subtypes.

A total of 60 participants [IBS-C (*n* = 27, 45%), IBS-D (*n* = 18, 30%), and IBS-M (*n* = 15, 25%)] received the six-month home FMT intervention during the study period (Figure 1). The median age at the commencement of treatment for the total cohort was 45 years (range: 18–73) with no significant differences observed between the three IBS subtype groups (Table 2). There was a greater number of females (*n* = 42, 70%) within the study cohort, consistent across all three IBS subtypes (Table 2). No significant differences in comorbidities of both coeliac disease and thyroid disorders; hypothyroidism and hyperthyroidism were observed between IBS-C, IBS-D, and IBS-M, and were present in 8% (*n* = 5) and 10% (*n* = 6) of the total cohort, respectively (Table 2). The most common pre-treatment antibiotics prescribed were vancomycin and rifaximin, used in 83% (*n* = 50) and 60% (*n* = 36) of participants, respectively (Table 2). No pre-treatment antibiotic combination was significantly over-represented within a specific IBS subtype (Table 2).

**Figure 2:**
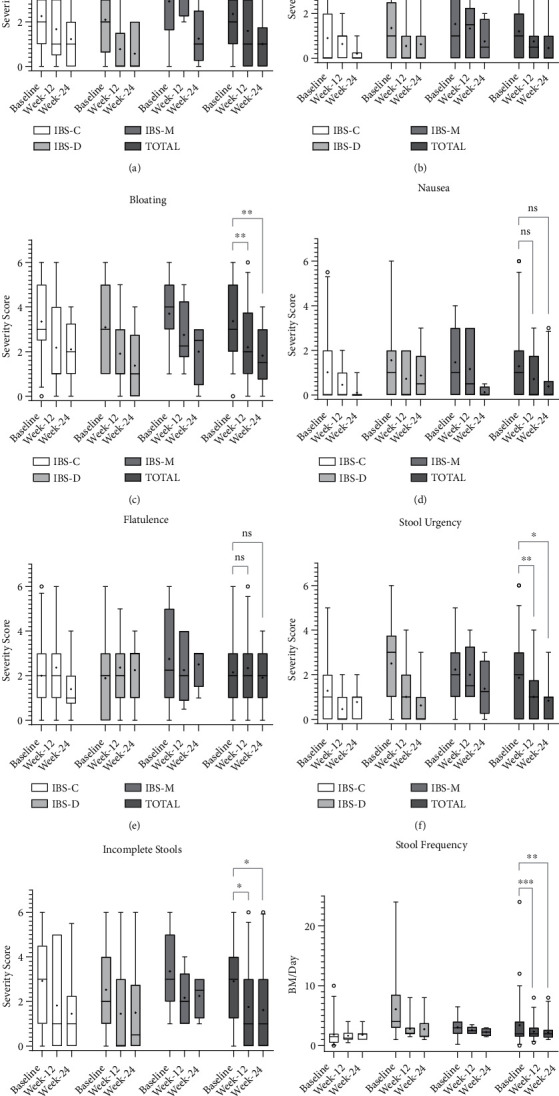
Abdominal symptom severity scores by IBS subtype and total cohort from baseline to week-24 (*N* = 59). Scores of abdominal symptom severity for (a)–(g) were as follows; 0 = none, 1 = mild, 2 = moderate, 3 = quite a lot, 4 = severe, 5 = very severe, and 6 = unbearable. Stool frequency (h) was recorded as bowel motions (BM) per day. A 5–95 percentile box and whisker plots (a)—(h) were generated in GraphPad Prism 8 with the median and quartiles marked as standard, the mean marked with a ‘+' and outliers marked with a ‘o'. Significance was graphed as follows; ns = not significant (*p* > 0.05), ∗*p* ≤ 0.05, ∗∗*p* ≤ 0.01, and ∗∗∗*p* ≤ 0.001. Week-52 data was omitted due to low questionnaire return rate.

A total of 59 (98%) participants [IBS-C (*n* = 27, 46%), IBS-D (*n* = 17, 29%), and IBS-M (*n* = 15, 25%)] returned baseline abdominal symptoms and SF-36 QOL questionnaires (Figure 1; Tables 3 and 4). Symptoms of bloating, stool urgency, and stool completeness were the most severe symptoms reported at baseline, reported in up to 98% of respondents, each with a median score of 3, indicative of moderate to severe severity [Table 3; Figures 2(c), 2(f), and 2(g)]. Conversely, heartburn (median score = 1, range 0–6) and nausea (median score = 1, range 0–6) were the two mildest symptoms reported at baseline, with almost half of participants reporting their absence [Table 3; Figures 2(b) and 2(d)]. Unsurprisingly, participants diagnosed with IBS-D reported significantly greater severity in stool urgency (median score = 3, range 0–6) compared to participants with IBS-C (median score = 1, range 0–5; *p* = 0.03), whilst participants with IBS-C reported a significantly lower number of daily bowel motions (median score = 1.5, range 0–10) compared with those participants in IBS-D (median score = 4, range 1–24) and IBS-M (median score = 3, range 0–7) subtypes (*p* < 0.0001; Figure 2). No significant difference in the severity of abdominal pain, heartburn, bloating, nausea, flatulence, and stool completeness was observed between the three IBS subtypes at baseline.

**Figure 3:**
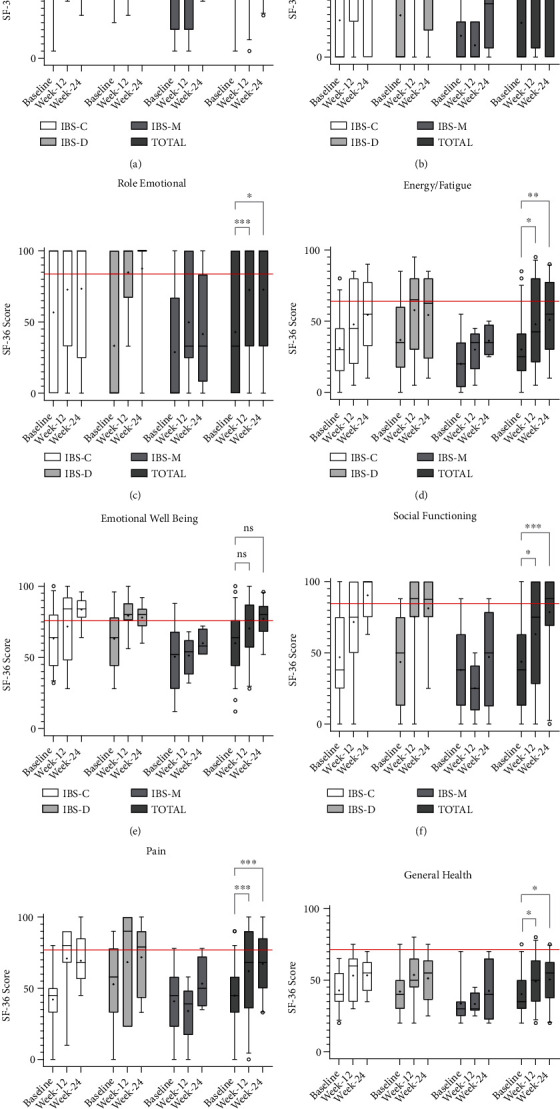
QOL component scores by IBS subtype and total cohort from baseline to week-24 (*N* = 59). SF-36 QOL component (a)—(h) scores are calculated from specific questions as a total percentage, with scores of 100 being the most positive and scores of zero being the most negative [47]. A 5–95 percentile box and whisker plots (a)—(h) were generated in GraphPad Prism 8 with the median and quartiles marked as standard, the mean marked with a ‘+' and outliers marked with a ‘o'. The addition of the red horizontal line is indicative of the average score for a healthy Australian individual (*a* = 83.6, *b* = 79.7, *c* = 83.7, *d* = 63.8, *e* = 75.7, *f* = 84.6, *g* = 76.9, and *h* = 71.5) [52]. Significance was graphed as follows; ns = not significant (*p* > 0.05), ∗*p* ≤ 0.05, ∗∗*p* ≤ 0.01, and ∗∗∗*p* ≤ 0.001. Week-52 data was omitted due to low questionnaire return rate.

Mean scores for all eight QOL components were significantly lower within our cohort at baseline when compared to the healthy Australian population means (*p* < 0.0001; Table 4; Figure 3). General health and vitality were amongst the lowest scored QOL components when compared to the healthy Australian norms, with 98% and 93% of baseline respondents reporting scores lower than the healthy Australian mean, respectively (Table 4). There were no significant differences in the eight components of the SF-36 QOL questionnaire at baseline between IBS subtypes.

### 3.2. Week-12 Outcomes

#### 3.2.1. Abdominal Symptoms

A total of 28 (47%) participants [IBS-C (*n* = 11, 39%), IBS-D (*n* = 11, 39%), and IBS-M (*n* = 6, 21%)] provided abdominal symptoms and SF-36 QOL questionnaires at week-12 (Figure 1; Tables 3 and 4). Median severity scores for all abdominal symptoms were found to have improved during the first 12 weeks of treatment with up to 61% of respondents reporting a reduction in severity when compared to baseline [Table 3; Figures 2(a)–2(h)]. Statistical significance was achieved in symptoms of bloating, stool urgency, stool completeness, and daily bowel motions [Figures 2(c), 2(f)–2(h)]. IBS subtype variations in abdominal symptom severity at week-12 were present, where participants with IBS-D returned significantly lower abdominal pain severity scores (median score = 0, range 0–4) compared to those with IBS-M [median score = 3.5, range 2–4; *p* = 0.02; Figure 2(a)].

#### 3.2.2. SF-36 QOL

Participants also reported notable improvements in QOL at week-12, where up to 71% of respondents achieved an increase in QOL score from baseline following 12 weeks of FMT (Table 4). Statistically significant increases in participant QOL was observed in all SF-36 QOL components except emotional well-being, which showed the lowest participant percentage improvement (43%) of all components from baseline [Table 4; Figures 3(a)–3(h)]. At week-12, no significant differences between respondent scores and the healthy Australian norms were observed in three QOL components, such as physical functioning, emotional role, and emotional well-being. The remaining five QOL component scores, however, remained significantly lower in week-12 respondents when compared to the healthy Australian norms despite all achieving statistically significant improvements from baseline (Table 4; Figure 3). Differences by IBS subtype was also observed within the SF-36 QOL questionnaire. Indeed, the significant improvements observed within respondents at week-12 for physical role, social functioning, and general health were significantly associated with participants from both IBS-C or IBS-D subtypes compared to participants with IBS-M; physical role (*p* = 0.005), social functioning (*p* = 0.02), and general health [*p* = 0.02; (Figures 3(b), 3(f), and 3(h)].

### 3.3. Week-24 Outcomes

#### 3.3.1. Abdominal Symptoms

A total of 22 (37%) participants [IBS-C (*n* = 10, 45%), IBS-D (*n* = 8, 36%), and IBS-M (*n* = 4, 18%)] provided abdominal symptoms and SF-36 QOL questionnaires at week-24 (Figure 1; Tables 3 and 4). All eight abdominal symptoms were found to have improved during the 6-month treatment period [Table 3; Figures 2(a)–2(h)], with the most significant improvements observed in bloating (median score = 1.5, range 0–4), where 64% of week-24 respondents reported a decline in severity from baseline [Table 3 and Figure 2(c)]. The significant reductions in the severity of bloating, stool urgency, stool completeness, and daily bowel motions observed in week-12 respondents were maintained through to the week-24 timepoint, where all remained significantly lower in severity when compared to matched baseline scores ([Figures 2(c), 2(f)–2(h)]. In addition, although the frequency of FMT infusions declined between week-12 and week-24 (Table 1), both heartburn and abdominal pain; which showed no significant reductions in severity at week-12, demonstrated a significant decline in severity in week-24 respondents [Figures 2(a) and 2(b)]. Conversely, despite an increasing percentage of participants reporting reductions in symptom severity from baseline across weeks 12 and 24, both nausea and flatulence showed no statistically significant change throughout the 6-month FMT treatment [Table 3 and Figures 2(d) and 2(e)]. At week-24, no significant differences between IBS subtypes were found.

#### 3.3.2. SF-36 QOL

Similar to abdominal symptoms, the improvements observed in QOL at week-12 were both maintained and further enhanced at week-24, where all SF-36 QOL components except for emotional well-being displayed significant increases in up to 76% of respondents compared to baseline scores [Table 4; Figures 3(a)–3(h)]. Physical functioning, emotional role, emotional well-being, social functioning, and pain, all were statistically indistinguishable from the healthy Australian norms at week-24, whereas 68% of respondents reported scores above the healthy mean for both physical functioning and emotional role [Table 4; Figures 3(a), 3(c) and (e)–(g)]. Week-24 scores for the remaining three QOL components, such as physical role, vitality, and general health remained significantly lower than the healthy Australian norms despite all showing significant increases from baseline [Figures 3(b), 3(d), and (h)]. IBS subtype variations in QOL improvements were also observed at week-24, where participants diagnosed with IBS-C reported significantly higher scores in emotional well-being (median score = 84, range 64–96; *p* = 0.009) and social functioning (median score = 100, range 63–100) compared to participants with IBS-M [(median score =58, range 52–72) and (median score = 50, range 0–88), respectively; *p* = 0.04; Figures 3(e) and 3(f)].

### 3.4. Mid-Treatment Prescriptions

In cases of limited or no response to FMT treatment (*n* = 16, 27%), participants were treated accordingly but not removed from the study. Eighteen prescriptions [olsalazine (*n* = 10) and colchicine (*n* = 8)] were provided to 16 participants during the 6-month FMT treatment period for their anti-constipation effects [53, 54]. Notably, participants with IBS-C were more likely to require such additional interventions during FMT (*n* = 16 prescriptions), with a statistically significant proportion receiving either olsalazine or colchicine (*p* = 0.0004) compared to IBS-D (*n* = 0) and IBS-M (*n* = 2) subtypes.

### 3.5. Week-52 Outcomes

#### 3.5.1. Abdominal Symptoms

A total of 14 (23%) participants [IBS-C (*n* = 5, 36%), IBS-D (*n* = 5, 36%), and IBS-M (*n* = 4, 28%)] provided abdominal symptoms and SF-36 QOL questionnaires at week-52 (Figure 1; Tables 3 and 4). Of these 14 participants, 11 (79%) had remained off all therapy following the cessation of FMT at week-24 up until the week-52 follow up. The remaining three participants continued the added olsalazine or colchicine therapy they received throughout the six-month FMT treatment. The median score scores for all abdominal symptoms were reduced at week-52, with up to 75% of respondents reporting symptom severity reduction from baseline (Table 3). Symptoms of heartburn and stool urgency displayed improvement from baseline in 43% and 57% of week-52 respondents, respectively (Table 3). Both nausea and flatulence trended towards severity reduction throughout the entire treatment and follow-up period (Table 3). Variation by IBS subtype could not be ascertained due to the lack of questionnaires returned at week-52.

#### 3.5.2. SF-36 QOL

Participant QOL at week-52 showed similar positive trends to abdominal symptoms. Increases in median scores for all SF-36 QOL components was seen at week-52, with up to 64% of respondents reporting an increase in QOL from baseline (Table 4). Emotional well-being showed no improvement across the entire follow-up period despite less than one third of respondents reporting scores below the healthy norms at week-52 compared to over two thirds at baseline (Table 4). Differences within IBS subtypes could not be ascertained due to the low rate of questionnaire respondents at week-52.

### 3.6. Six-Month FMT Treatment Completion

A total of 11 (18%) participants did not complete the six-month FMT treatment. Of these 7 (64%) failures were a result of home donor-related issues ranging from donors contracting infections that required the use of antibiotics, to the donor moving away from the participant during the treatment period. The remaining four participants failed to complete the entire six-month treatment due to adverse events such as vomiting, nausea, suicidal ideation, and an anal fissure caused by the insertion of the enema catheter. Whether the incidence of vomiting and nausea as well as the single case of suicidal ideation were directly caused by FMT remains speculative. Due to the small number of participants who did not complete the six-month FMT treatment, no sub-group analysis of the outcomes of those participants who completed the entire treatment versus those who only completed a proportion of it could be performed.

### 3.7. Adverse Events

Overall, 25 adverse events were reported in 17 (28%) participants with gastrointestinal symptoms being the most common (*n* = 9, 36%) followed by various infections (*n* = 7, 19%; Table 5). Of note, none of the 25 adverse events were graded above two on the CTCAE severity scale, suggesting that they were both transient and mild in nature (Table 5). The single incidence of transient *Campylobacter-*associated gastroenteritis was acquired from a home donor prior to symptom onset. There were four reported cases of thrush, one oral and three vaginal. The single case of suicidal ideation was pre-existing, however, at the time of recurrence, the participant was removed from the six-month FMT treatment program to obtain appropriate psychological intervention. Two surgical and medical procedure adverse events were caused by the enema catheters and both occurred whilst the participants were performing FMT infusions at home. One participant failed to remove the enema spigot cap before insertion, resulting in it becoming lodged within the rectum, requiring medical assistance for safe removal. The other case, also caused by the enema spigot, was a minor anal fissure caused by participant insertion that required treatment and cessation of further FMT infusions.

## 4. Discussion

To the best of our knowledge, this is the first study to report a pre-antibiotic treatment and sustained six-month enema FMT program for the treatment of IBS or, of any disorder. The findings suggest that pre-antibiotic treatment and six months of sustained FMT, beginning with a single colonoscopic infusion followed by a series of retention enemas is a safe and effective treatment for IBS that improves both abdominal symptoms and QOL for up to six months post-FMT treatment. Reduced severity in abdominal symptoms was seen in up to 64% of participants during the six-month FMT treatment. These included significant reductions in the severity of abdominal pain, bloating, and improvements in stool completeness and stool frequency, which were maintained up to six months post-FMT without additional therapy. Likewise, improvements in QOL were seen in up to 76% of participants during FMT treatment with five out of eight QOL components showing no statistical difference from the healthy Australian norms at the conclusion of FMT. Significant improvements in physical role, social functioning, and general health QOL components were maintained at the 52-week follow up. Adverse events were reported in 28% of participants, though they were both transient and mild in nature, causing only 7% of participants to cease FMT before the end of the six-month treatment period. Of those, only 3% of adverse events were directly related to home infusions.

Several other open-label SATs [20–23] and larger, more robust RCTs [24–30] which aimed to assess the therapeutic utility of FMT in IBS have also been conducted. These studies used one infused FMT per treatment or up to 225 lyophilised FMT capsules over three days. In comparison, our novel 6-month repeated FMT protocol offers a unique application of extended FMT treatment. Compared to published SATs, where 60%–75% of participants reported symptomatic improvement, our extended FMT protocol showed similar short-term percentage improvement in both IBS related abdominal symptoms and QOL [20–23]. Likewise, in three RCTs which report greater efficacy of FMT compared to placebo, short-term symptomatic improvement was achieved in 56%–89% of participants, suggesting no clear advantage for repeated FMT in short-term symptom severity reduction [24, 26, 27]. The long-term maintenance of symptom improvement, however, has seldom been a principal outcome in studies utilising single FMT treatments. Indeed, only two SATs [20, 23] and four RCTs [25, 26, 30, 55] have reported on the longevity of symptom improvements after FMT. Importantly, out of these six studies, two reported deteriorating response rate of up to 63% one year after FMT [20, 26], two reported a range of 78%–88% of respondents achieving sustained long-term improvement one year after FMT [23, 55] and the remaining two found no significant short-term improvements, thus limiting long-term efficacy assessment [25, 30]. Two studies have also reported significant improvement in the short term and long term of IBS symptoms and changes to the gut microbiota [56, 57].

Here, we report the maintenance of short-term symptomatic improvement in 75% of week-52 respondents, 6 months after their final FMT, suggesting that a prolonged FMT treatment regime may result in the long-term treatment free alleviation of IBS associated symptoms, leading to improved QOL.

As previously mentioned, to the best of our knowledge only two other studies have reported the maintenance of symptom improvement for six months or more after FMT. Interestingly, one of these studies, conducted by El-Salhy et al. [55] reports on the use of a single donor capable of inducing near 90% long-term improvement in IBS patients following the infusion of one FMT into the distal duodenum via gastroscope. The authors suggest that the high rates of efficacy achieved in their study was due to the recently proposed “super donor” phenomenon [58]. Indeed, traditional FMT screening criteria focuses heavily on recipient safety with limited to no assessment of donor quality [39, 40]. Hence, future studies utilising additional screening criteria known to affect the composition of the gut microbiome such as sleep quality, stress, physical activity, diet and overall quality of life may yield more efficacious donors, leading to improved clinical response in chronic conditions such as IBS and IBD. However, with donor qualification rates under the current screening criteria already as low as 2.5% [59], adding additional efficacy related standards would likely hinder the scope of its application.

Several novel aspects of the FMT protocol reported here, which aimed to increase the implantation of donor taxa, are possible contributors to the long-term improvement achieved within our cohort. Firstly, in comparison with previously published literature on FMT in IBS, our regime involves the infusion of approximately 25 times the volume of donor faecal material. Other studies have typically either utilised a single 30–100 g FMT infusion, delivered into the caecum or duodenum [20–27], or in the case of three RCTs, a 12-day treatment of orally encapsulated FMT, totalling approximately 100–150 g of freeze-dried donor stool [28–30]. Comparatively, our novel protocol entails the infusion of approximately 75 g of donor stool per 300 ml homogenate infusion, which across the six-month protocol equates to roughly 2.4 kg of donor faecal material (Table 1). Secondly, in conjunction with the sustained six-month FMT program, another novel aspect of the protocol reported here was the use of pre-treatment antibiotics. To the best of our knowledge, the use of antibiotics prior to FMT treatment in IBS has not yet been reported despite evidence from studies on other gastrointestinal conditions suggesting that its usage can aid in the implantation of beneficial taxa, such as *Bifidobacterium*, known to be deficient in patients with IBS when compared with healthy controls [34, 60–62]. Moreover, the addition of the three prebiotic fibre supplements, such as apple pectin, inulin, and NAG, used throughout the six-month treatment period are known to promote the production of SCFAs, may have also encouraged the implantation of beneficial taxa from donor faecal material and therefore assisted in achieving a sustained therapeutic effect [41, 42, 63]. Future studies on FMT in IBS should aim to establish whether pre-treatment antibiotics, repeated FMT infusions and high dietary fibre intake are associated with increased donor microbiota implantation, resulting in prolonged improvement.

Both the rate and severity of adverse events reported in our cohort are analogous to those reported in previously published literature [64, 65]. Indeed, one systematic review published in 2016 reported pooled adverse event rates of 20.6% and 49.3% for lower and upper gastrointestinal routes of FMT, respectively [64]. Here, we report an adverse event rate of 28%, suggesting that our novel extended FMT protocol, where the majority of treatment was performed by the participants themselves, does not increase the rate or the severity of adverse events compared to single FMT infusions or encapsulated FMT performed in the clinic. Our results suggest that our novel home FMT protocol was well tolerated and accepted by participants as significant increases in the majority of QOL components were observed whilst participants were receiving treatment. In addition, a separate study conducted in 2018 reported that 97% of individuals who had previously performed FMT themselves would do it again [66]. Notable adverse events attributable to the home FMT component of the protocol used in this study included the two reported surgical and medical procedure-related adverse events. The low incidence (3%) within the total cohort suggests that the single training session with a qualified nurse and continued support throughout the six-month treatment was sufficient in minimising procedural related adverse events whilst participants were performing FMT infusions themselves. Importantly, as no increase in the rate of adverse effects attributable to FMT were observed, we suggest that this novel protocol may be applicable in other chronic gastrointestinal diseases such as IBD which have also shown improved FMT efficacy following multiple FMT infusions [36–38].

Response variation by IBS subtype in both abdominal symptoms and QOL outcomes was yet another interesting finding from this study. We observed that participants diagnosed with IBS-M tended to report QOL improvement to a lesser degree at both weeks 12 and 24 compared to both other subtypes and that those diagnosed with IBS-C required additional adjunct therapy to achieve the extent of symptomatic improvement those diagnosed with IBS-D obtained when treated with FMT alone. The authors acknowledge that due to the significant reduction in participant questionnaire response over time, the IBS subtype differences identified here are somewhat unreliable due to the inherent limitations of sample bias. However, to the best of our knowledge, IBS subtype variation in response to FMT to this degree has not previously been reported and may highlight possible different pathological pathways responsible for the manifestation of each IBS subtype.

Despite its novelty, this study is not without limitations. Firstly, this was an observational, clinical study, hence the protocol was individualised to the individual and no placebo or control group was available. Secondly, in order to capture a wider range of symptoms and their severity changes throughout treatment with FMT, we utilised an alternative abdominal symptom-based questionnaire which is not commonly used elsewhere [48], therefore reducing the comparability of the results reported within this study. In addition, the timing of the baseline questionnaire were not reflective of a true pre-treatment condition in participants since antibiotics, and in particular, rifaximin, are known to be effective treatments for IBS [67] and also antibiotics, prebiotics, and bowel cleansing could have contributed to the symptom improvement. A further limitation of this study was the omission of faecal microbiota genetic sequencing from the study protocol, therefore, limiting the extent to which the significant symptomatic and QOL improvements achieved both throughout treatment and beyond can be associated with the implantation and maintenance of healthy donor microbiota. Finally, the loss of participant questionnaire response rate observed across the study period was notable and may have promoted response bias.

In summary, our findings suggest that in the clinical setting multiple FMT infusions over a period of six months successfully reduces the severity of abdominal symptoms associated with IBS, and significantly improves the QOL when compared to healthy Australian norms. We also report that the maintenance of these improvements six months after the cessation of FMT in up to 75% of respondents, suggesting that multiple FMT infusions may be one way to increase the long-term efficacy of FMT in IBS. Our results indicate that these improvements were achieved to a greater extent by participants with IBS-D when compared to participants diagnosed with either IBS-M or IBS-C, who may require additional pharmacological or psychological therapy whilst being treated with FMT to achieve similar outcomes. We also report for the first time, a safe, six months sustained FMT infusion protocol that allows participants to complete the majority of treatment whilst at home, which may be applicable in other chronic gastrointestinal diseases that show increased efficacy with repeated FMT dosing.

## Figures and Tables

**Table 1:** Six-month FMT schedule.

Weeks of treatment	No. of infusions per week	Infusion techniques	FMT volume infused per week (ml)	Donor	Location performed
1–2	5	Colonoscopic infusion (day 1) and rectal retention enemas (days 2–10)	1500	Pooled centre donors	Centre
Home FMT education session					Centre
3	4	Rectal retention enemas	1200	Home donors	Home
4	3	Rectal retention enemas	900	Home donors	Home
5	2	Rectal retention enemas	600	Home donors	Home
6–11	1	Rectal retention enemas	300	Home donors	Home
12–24	0.5†	Rectal retention enemas	150	Home donors	Home

^†^300 ml infused once fortnightly, not 150 ml per week.

**Table 2:** Baseline demographics of the participants.

Characteristics	IBS-C (*n* = 27)	IBS-D (*n* = 18)	IBS-M (*n* = 15)	Total (*n* = 60)	*p*-Value
Age (median)	40	48	39	45	0.3
Sex (*n* female)	20	13	9	42	0.6
Comorbidities					0.5
Coeliac disease (*n*)	4	0	1	5	
Thyroid disorder (*n*)	2	2	2	6	
None (*n*)	21	16	12	49	
Pre-treatment antibiotic combination					0.1
Vancomycin + rifaximin (*n*)	12	5	7	24	
Vancomycin alone (*n*)	9	4	5	18	
Other† (*n*)	6	9	3	18	

^†^Other = vancomycin + tinidazole (*n* = 5), rifaximin + tinidazole (*n* = 4), rifaximin alone (*n* = 3), vancomycin + rifaximin + tinidazole (*n* = 2), rifaximin + metronidazole (*n* = 2), vancomycin + rifaximin + metronidazole (*n* = 1), and tinidazole alone (*n* = 1).

**Table 3:** Descriptive summary of abdominal symptom scores across baseline, week-12, week-24, and week-52 (*N* = 59).

Component	Timepoint	Component respondents *N*	Absence of symptoms† *N* (%)	Severity reduction from baseline *N* (%)	Severity increase from baseline *N* (%)
Abdominal pain	Baseline	55	8 (15)		
	Week-12	22	9 (41)	11 (50)	5 (23)
	Week-24	20	9 (45)	11 (55)	3 (15)
	Week-52	12	7 (58)	9 (75)	1 (8)
Heartburn	Baseline	58	25 (45)		
	Week-12	28	14 (50)	11 (39)	5 (18)
	Week-24	22	15 (68)	9 (41)	1 (5)
	Week-52	14	8 (57)	6 (43)	3 (21)
Bloating	Baseline	59	1 (2)		
	Week-12	28	3 (11)	14 (50)	5 (18)
	Week-24	22	5 (23)	14 (64)	5 (23)
	Week-52	14	2 (14)	8 (57)	2 (14)
Nausea	Baseline	59	28 (47)		
	Week-12	28	17 (61)	8 (29)	3 (11)
	Week-24	22	16 (73)	7 (32)	3 (14)
	Week-52	14	9 (64)	5 (36)	3 (21)
Flatulence	Baseline	57	11 (19)		
	Week-12	28	2 (7)	11 (39)	12 (43)
	Week-24	22	3 (14)	7 (32)	7 (32)
	Week-52	14	1 (7)	5 (36)	7 (50)
Stool urgency	Baseline	57	15 (26)		
	Week-12	28	12 (43)	17 (61)	6 (21)
	Week-24	21	9 (43)	11 (52)	3 (14)
	Week-52	14	4 (29)	8 (57)	2 (14)
Incomplete stools	Baseline	56	6 (11)		
	Week-12	28	11 (39)	15 (54)	5 (18)
	Week-24	22	8 (36)	13 (59)	3 (14)
	Week-52	14	6 (43)	8 (57)	1 (7)

^†^A score of 0 which correlates to the absence of the associated symptom as described in Figure 2 legend. Stool frequency data is presented only in Figure 2.

**Table 4:** Descriptive summary of short form-36 quality of life scores across baseline, week-12, week-24, and week-52 (*N* = 59).

Questionnaire component	Timepoint	Component respondents *N*	Below healthy norm† *N* (%)	QOL increase from baseline *N* (%)	QOL decrease from baseline *N* (%)
Physical functioning	Baseline	58	30 (52)		
	Week-12	27	10 (37)	16 (59)	5 (19)
	Week-24	22	7 (32)	14 (64)	4 (18)
	Week-52	14	6 (43)	9 (64)	4 (29)
Physical role	Baseline	59	50 (85)		
	Week-12	28	15 (54)	14 (50)	2 (28)
	Week-24	22	11 (50)	9 (41)	0 (0)
	Week-52	14	6 (43)	7 (50)	1 (7)
Emotional role	Baseline	59	37 (63)		
	Week-12	28	11 (39)	14 (50)	0 (0)
	Week-24	22	7 (32)	9 (41)	1 (5)
	Week-52	14	6 (43)	7 (50)	2 (14)
Vitality	Baseline	58	54 (93)		
	Week-12	28	18 (64)	18 (64)	8 (29)
	Week-24	21	14 (67)	15 (71)	6 (29)
	Week-52	14	8 (57)	9 (64)	3 (21)
Emotional well-being	Baseline	59	41 (69)		
	Week-12	28	13 (46)	12 (43)	11 (39)
	Week-24	21	8 (38)	12 (57)	7 (33)
	Week-52	14	4 (29)	8 (57)	5 (36)
Social functioning	Baseline	59	49 (83)		
	Week-12	28	16 (57)	16 (57)	4 (14)
	Week-24	21	10 (48)	14 (67)	1 (5)
	Week-52	14	8 (57)	9 (64)	2 (14)
Pain	Baseline	59	49 (83)		
	Week-12	28	16 (57)	20 (71)	6 (21)
	Week-24	21	12 (57)	16 (76)	2 (10)
	Week-52	14	7 (50)	8 (57)	2 (14)
General health	Baseline	59	58 (98)		
	Week-12	28	25 (89)	16 (57)	8 (29)
	Week-24	21	20 (95)	12 (60)	5 (24)
	Week-52	14	14 (100)	7 (50)	1 (7)

^†^Healthy norms were taken from the Australian Institute of Health and Welfare Report. [52] Healthy mean scores are listed in Figure 3 legend.

**Table 5:** Common terminology criteria for adverse events (CTCAE) grading of participant reported adverse events during FMT (*N* = 60).

Adverse event	Severity grade†					
	One *N*	Two *N*	Three *N*	Four *N*	Five *N*	Total *N*

Gastrointestinal disorders						
						
Bloating		3				3
						
Nausea	1	3				4
						
Vomiting	2					2
						
General disorders and administration site conditions						
						
Fatigue	1					1
						
Flu-like symptoms	1					1
						
Infections and infestations						
						
Mucosal infection *(oral thrush)*	1					1
						
Sinusitis		1				1
						
Small intestine infection		1				1
						
Urinary tract infection		1				1
						
Vaginal infection *(thrush)*		3				3
						
Nervous system disorders						
						
Headaches	2	1				3
						
Psychiatric disorders						
						
Suicidal ideation		1				1
						
Respiratory, thoracic, and mediastinal disorders						
						
Sore throat	1					1
						
Surgical and medical procedures						
						
Anal fissure repair		1				1
						
Rectal foreign body removal		1				1
						

^†^Severity grade refers to the severity of the adverse event. Grade 1: mild; asymptomatic or mild symptoms; clinical or diagnostic observations only; intervention not indicated. Grade 2: moderate; minimal, local, or non-invasive intervention indicated; limiting age-appropriate instrumental activities of daily living. Grade 3: severe or medically significant but not immediately life-threatening; hospitalization or prolongation of hospitalization indicated; disabling; limiting self-care activities of daily living. Grade 4: life-threatening consequences; urgent intervention indicated. Grade 5: death related to adverse events [49].

## Data Availability

Data was analysed from the secure practice clinical server and history records. The data that support the findings of this study are available from the Centre for Digestive Diseases, but restrictions apply to the availability of these data, which were used under license for the current study, and so are not publicly available. Data are however available from the authors upon reasonable request and with permission of the Centre for Digestive Disease.
